# The Practical Utility of Dentistry

**Published:** 1860-10

**Authors:** A. J. Brown


### ARTICLE V.

### *The Practical Utility of Dentistry.*

By A. J. Brown, D. D. S.

It is sometimes said that we are living under a curse, that the judicial sentence of the Creator which has consigned the teeming millions of Adam's race to a condition of toil imposing upon them the necessity of earning their daily bread in the sweat of their face, is felt to be burdensome, if not oppressive. But this sentiment is generally found only with the unreflecting, for what would be the spectacle presented if the various trades, callings and professions which now give employment and the means of subsistence to civilized men, were to be abolished, and means of supporting life without involving the necessity of labor, should be put within the reach of every man? Who does not see that it would be followed immediately by a rapid degeneration of the human race? Anarchy would reign in the place of law and order. Government, in all its departments,—in the state, the church and the family, would give place to the dominion of mere brute force; purity and refinement would flee before the unrestrained power of sensuality, and so rapid would be the retrogression of education, science and the arts, that only a very brief period would be required to reduce the most enlightened nation on earth to a condition worse than any barbarism known in the annals of the world. What, but the *necessity of labor* prevents us from calamities so great?—That necessity, then, while it results from a judicial sentence of the Creator, is among the most merciful provisions for the well being of man.

Among the various callings and professions which have originated in the necessities of the human race, there is to be seen a great diversity, both as regards the degree of perfection to which they have attained, and the purposes of utility which they are intended to serve. I propose, in this paper, to call attention to the ample sphere of usefulness which is afforded by our profession, with a view to setting forth the benefits we dispense, and of showing upon what insufficient grounds the opinion rests that ours is an unimportant calling—an opinion more than expressed when it is said of any one of our brethren, "*O! he is only a dentist!*"

There are to be found in every profession and calling, men who are actuated only by selfish motives. To supply their own necessities and provide for their own enjoyment in life, are the only objects which stimulate them to study or labor. It follows, therefore, that so far from proving a source of daily pleasure to them, their employment must be found irksome and burdensome, and the time devoted to them a period of unhappy slavery. But when from the outset the aim of the practitioner embraces the good of others, and experience has taught him to appreciate the luxury of doing good, he finds his duties and employments a *pleasure* which not only reconciles him to whatever burdens they may impose, but incites him to enthusiasm and inclines to patient study and toil, with a view of advancing to perfection in the art he loves. Looking beyond the limits of self-interest and the sphere of self-enjoyment, to the purer and nobler field where philanthropy dispenses its blessings and man lights up the sorrowful eye of his fellow man, relieves him of his burdens, mitigates his afflictions, and pours the oil and wine of comfort and peace into his stricken bosom, the dentist as well as the physician beholds his unselfish mission, and devotes himself to the amelioration of the condition of his fellow beings. In an object so noble, he will find enough to stimulate him to industry and application, in the developing and perfecting of all the resources of our art. He will study to acquaint himself with all those principles of philosophy, surgery, and laws of chemistry which the science of dentistry involves, and a knowledge of which is indispensable to its pursuit and its advancement to that perfection to which it is ultimately destined to attain.

But while in his office or laboratory, the practitioner of dentistry applies himself with patience and industry, to the duties of his profession, his labors will be attended with only limited results, unless the benefits they are intended to confer are known and appreciated in the world. Hence the aid of the press, must, in this, as well as in all other subjects of importance to the well being of the public, be invoked. Through that powerful agency, our profession must seek to inform the public mind of its humane objects, and of the constantly increasing means it possesses for their accomplishment.

It is perhaps true to a degree but little suspected, that there is a lamentable ignorance prevailing on this subject —that comparatively a few, only, are aware of the importance of a healthy, well managed denture, or of the various causes which produce, and are constantly producing effects not only upon the teeth, but the general health, which are extremely deleterious.

Does any one ask for a proof? He has it abundantly supplied in the common practice so very prevalent among the young and the less informed, of using their teeth as *nut crackers*, or crushing other hard substances, obviously contrary to the intentions of nature. He has it in the less apparently, but equally pernicious, habit of partaking of ices at one moment, and at the next of drinks on the other extreme of temperature, whereby are incurred the evils which result so disastrously from the subjection of the teeth to a sudden transition from cold to heat.

Perhaps it would not be very far from the truth to affirm that a majority of persons who give any attention to their teeth, are influenced by a consideration of beauty, rather than of utility; or at least they do not go beyond the object of securing a corrective for the otherwise unpleasant odor of their breath; and while we would by no means undervalue the importance of these objects, knowing the additional charm that beauty borrows from a regular, unbroken, spotless set of teeth, and the fact that applications for divorce have been based on the fetid breath of a conjugal companion, we would yet maintain that it is the province of the skillful dentist not only to supply the one, and correct the other, but to confer more valuable benefits, or, if you please, avert greater misfortunes. Need we adduce facts to fortify this assertion? The whole train of evils, beginning with calculous accumulations, sponginess of the gums, caries, abscesses, pain, nay excruciating, agonizing torture, convulsions, nervous excitement, delirium, compulsory fasting, exhaustion and prostration—and ending in the gradual but effectual destruction of the general health, or irremediably diseased condition of some of the vital organs, the stomach or the lungs, but all of which might be prevented by timely and judicious dental supervision, and operation—abundantly attest that amplitude of the field of usefulness which is open to the diligent and skillful dentist. But to all this might be added, the mental suffering, the irritation of temper, the loss of patience, which not only renders the patient miserable himself, but involves all others in unhappiness and disquietude, who come within the sphere of his presence. We feel perfectly safe, then, in saying that the degree of the influence of diseased teeth upon the health of the body, is neither known nor considered by the mass of our fellow creatures. It is a serious evil that information on this point is so limited, for this is not one of those cases in which the maxim —"where ignorance is bliss 'tis folly to be wise," is admissible. It would be as wise to neglect a gimlet hole in the bottom of a ship as to disregard the slightest indication of decay in a single tooth. Many of the diseases of the throat, so prevalent and so troublesome, are doubtless the result of decayed teeth, imparting disease to the mucous surfaces, which continues to defy all remedial agents, as long as the cause in the neglected teeth remains.

The necessity of proper ventilation to health, is now frequently made a theme for the scientific lecture, and the increased attention to this subject has verified the adage, "necessity is the mother of inventions." Many a piece of ingeniously constructed apparatus, and especially the ventilator but recently invented and patented by Mr. Colhoun of Philadelphia, attests the importance attached by scientific men, to purity of atmosphere. But why seek to ventilate your sleeping apartments, or leave the city, in sultry months, for the pure air of the country, while a cause is permitted to exist, which, at every respiration, conveys to the stomach and lungs, and from those organs to every part of the human body, a vitiated, infectious effluvia, which is as certain to interfere with the laws of health as the foul air confined in a ship or dwelling house, or the poisonous miasma which produces the congested and yellow fever of the South, by which thousands perish annually. It is a difficult task to produce a proper sense of the value of a sound and healthy mouth, in the mind of one who has never suffered the ills to which we have already referred. Only those whose lot it is to be afflicted or suffer from some obdurate or chronic disease, can appreciate the blessing of health. So those who are favored by nature, or have secured to them by the care and supervision of parents, a good set of teeth, can but imperfectly appreciate that benefit, or conceive of the life-long misery and discomfort which burdens the existence of those who through neglect or accident have lost their teeth, or have suffered them to fall into a diseased condition. How few, when pitying their friends suffering from the horrors of nervous excitability, neuralgia of the head and face, dyspepsia with all its train of mental and physical woes—conjecture the real cause, which, in innumerable cases, produces these painful and distressing symptoms! Bad teeth, retaining particles of food which decompose; imperfect mastication, rendering the process of digestion and assimilation uncertain and often impossible, though but little suspected, are frequently the productive cause of disease, which, accumulating in power, eventually consigns its victim to the grave—or what is worse, to the cell of the maniac.

Now, if to prevent such evils, and in a great measure remove them, by removing the cause by which they are produced, be the office of the skillful dentist, who can speak in terms of disparagement of his profession or his work? Nay, is he not, when combining the spirit of philanthropy and benevolence with the energy with which he prosecutes his business, justly entitled to be ranked among the benefactors of mankind? It has been said of one whose threescore and ten years have expired, that he has been heard to say, that, although in the course of his chequered life he had experienced a taste of nearly all the miseries usually incident to the present state of being—fortune and misfortune—wealth and poverty—yet he had suffered more real misery from the effects of bad teeth than from all other causes combined. Nor is this all.—This aged sufferer affirmed that had he been aware of the existence of the remedy at the proper time, and especially of the existence of the necessity of its application, he could have smiled upon all other evils with complacency. How would such a one, could he have the privilege of living his life over, with the benefit of his former experience, and his mind illuminated by the light of science, regard the dental art, and those of his fellow men who have devoted their lives to its advancement, and to the dispensation of the benefits which it proposes to confer on the race of man.

